# The complete mitochondrial genome of small narrow-mouthed frog, *Glyphoglossus yunnanensis* (Boulenger, 1919) (Amphibia: Anura: Microhylidae)

**DOI:** 10.1080/23802359.2022.2090293

**Published:** 2022-06-28

**Authors:** Shuang Huang, Yan Huang

**Affiliations:** a College of Life Science, Key Laboratory of Southwest China Wildlife Resources Conservation (Ministry of Education), China West Normal University, Nanchong, China; b College of Life Science, Institute of Eco-adaptation in Amphibians and Reptiles, China West Normal University, Nanchong, China

**Keywords:** Mitogenome, *Glyphoglossus*, China, Bayesian

## Abstract

The complete mitochondrial genome (mtDNA) of *Glyphoglossus yunnanensis* (Anura: Microhylidae) consists of a circular DNA molecule of 16,710 bp and encoded 13 protein-coding genes (PCGs), 22 transfer RNA (tRNA) genes, two ribosomal RNA (rRNA) genes, and non-coding regions of an L-strand replication origin and a D-loop region. All PCGs use ATN as the start codon, except for ND2, ATP8, ND4L, and Cytb uses the typical stop codon TAA/TAG; COI, ND6 use AGG; the other PCGs stop with a single T. The length of tRNAs ranged from 66 bp to 73 bp. The Bayesian phylogenetic analysis recovers *Glyphoglossus* and *Microhyla* as sister taxa, corroborating previous results.

The genus *Glyphoglossus* Günther, 1869 (Anura: Microhylidae) is a clade of fossorial frogs which mainly found in the range from Southern China to Indo-Malaya. Ten species of this genus have been found, two species recorded in China (Frost 2019; Zhang et al. 2021). *Glyphoglossus yunnanensis* Boulenger, 1919 mainly distributed in southwest China and north Vietnam which live in mountainous areas with an average elevation of over 1700 m, the adults breed in May (Fei et al. 2012). In this study, we present the first complete mitochondrial genomes of the genus *Glyphoglossus*.

The sample (SAMN20166212) of *G. yunnanensis* was collected in Wulong Fairy Mountain National Forest Park (29°27′25.87″N,107°42′45.51″E), Chongqing, China, at an altitude of 1795 m. The mtDNA sequences were obtained by next-generation sequencing (Illumina NovaSeq 6000; Sangon Biotech Co., Ltd., Shanghai, China) for PE 2 × 150 BP sequencing. Protein-coding genes (PCGs) and ribosomal RNA (rRNA) genes were mainly determined by alignment with the mitochondrial genomes of existing species *Microhyla heymonsi* (AY458596), *Kaloula rugifera* (KT878719), *Microhyla ornata* (DQ512876), and *Kaloula verrucosa* (MG962359) in GenBank using Geneious 11.0.2. Transfer RNA (tRNA) gene was predicted and determined by tRNAScan-SE server v 1.21 (Lowe and Eddy 1997) and MITOS WebSever (Bernt et al. 2013). The voucher specimen was deposited at the College of Life Science, China West Normal University (https://life.cwnu.edu.cn, Yan Huang and sunflower-hy@126.com).

The complete and circular mtDNA sequence is 16,710 bp in size. The overall nucleotide composition of this genome was 29.16% A, 27.03% C, 14.19% G, and 29.62% T, with a total A + T content of 58.76%. The sequence characteristic of A + T rich is similar to *Fejervarya limnocharis* and *Rana nigromaculata* (Sumida et al. 2001; Liu et al. 2005). Among the 37 mitochondrial genes, eight tRNA genes and ND6 genes were encoded by the L-strand, while the remaining genes including 12 PCG, 14 tRNA genes, and two rRNA genes were encoded by the H-strand. The absolute length of the 13 PCGs was 11,290 bp, with most PCGs beginning with a conventional ATG codon, except for COI with ATA; AGG was found as a stop codon in ND6 and COI, and TAG as a stop codon in ND2; TAA/TAG was found as a stop codon in ATP8, ND4L, Cytb, and ND1, COII, ATP6, COIII, ND3, ND4, and ND5 terminated with a separate T, apparently completed as TAA by post-transcriptional polyadenylation (Anderson et al. 1981). The size of the 22 tRNA masses changed from 65 bp to 73 bp. The two rRNA masses were 941 bp (12S) and 1583 bp (16S), respectively. Comparing *G. yunnanensis* with the 12 mitochondrial genomes identified in Microhylidae revealed that *Glyphoglossus*, *Microhyla*, and *Kaloula* mitochondrial genes are in the same order as in previous studies (Lin and Liu 2017).

Mitochondrial PCGs and 16S rRNA genes of 27 species were downloaded from NCBI in PhyloSuite (Zhang et al. 2020) and used for phylogenetic analyses. In PhyloSuite, Batch alignment of the 28 sequences was performed using MAFFT (Katoh and Standley 2013). Best parceling plan and developmental models for 28 pre-characterized allotments were chosen utilizing PartitionFinder2 (Lanfear et al. 2017), with covetous calculation and AICc criteria. A Bayesian inference phylogeny was derived using MrBayes 3.2.6 (Ronquist et al. 2012). The phylogenetic tree recovered *Glyphoglossus* as the sister taxon of *Microhyla* (Figure 1) corroborating previous phylogenetic studies (Matsui et al. 2011; Gorin et al. 2020).

**Figure 1.**
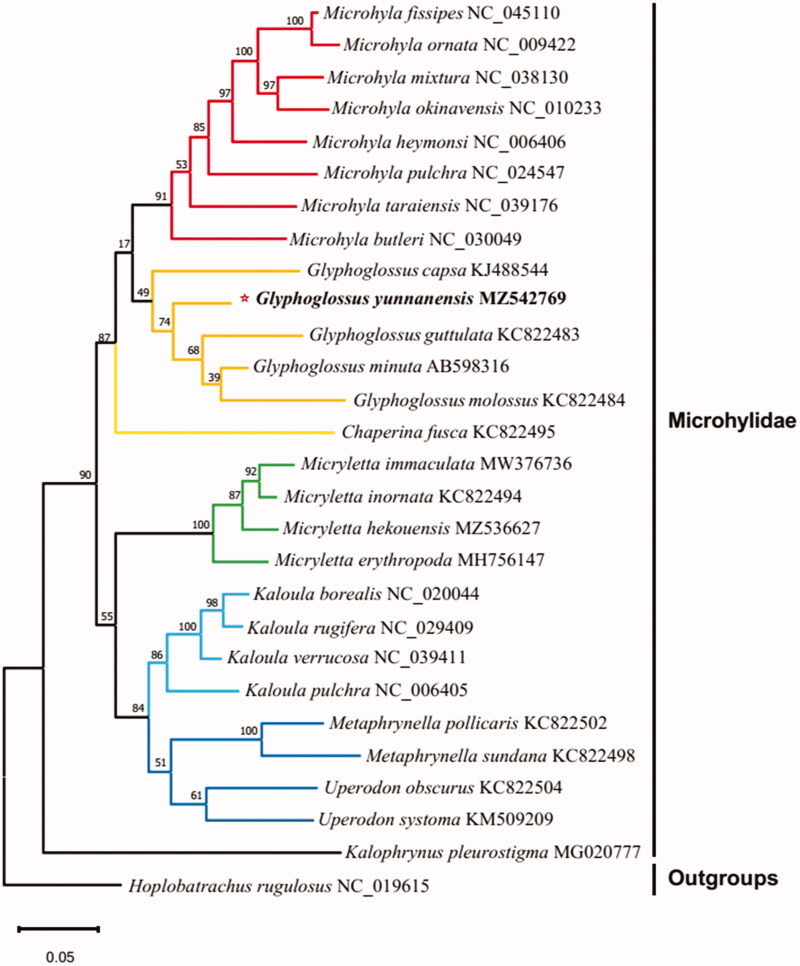
Bayesian phylogenetic tree of *G. yunnanensis* and other species of Amphibia based on 13 mitochondrial PCGs and 16S rRNA genes. *Hoplobatrachus chinensis* were selected as outgroups. Number nodes are bootstrap supports.

## Data Availability

The complete mitochondrial genome sequence of *Glyphoglossus yunnanensis* is deposited in the GenBank database under the accession number MZ542769 (https://www.ncbi.nlm.nih.gov/nuccore/MZ542769). The associated BioProject, SRA, and BioSample numbers are PRJNA745446, SRR15097471, and SAMN20166212, respectively.
